# Tribological Performance of CAM-Processed Interim Dental Restoration Materials: Effects of 3D Printing, Milling, and Post-Processing on Wear and Surface Topography

**DOI:** 10.3390/jfb17030136

**Published:** 2026-03-10

**Authors:** Liliana Porojan, Roxana Diana Vasiliu, Flavia Roxana Bejan, Mihaela Ionela Gherban, Diana Uțu, Anamaria Matichescu

**Affiliations:** 1 Department of Dental Prostheses Technology (Dental Technology), Center for Advanced Technologies in Dental Prosthodontics, Faculty of Dental Medicine, “Victor Babes” University of Medicine and Pharmacy Timișoara, Eftimie Murgu Sq. No. 2, 300041 Timișoara, Romania; sliliana@umft.ro (L.P.); flavia.toma@umft.ro (F.R.B.); 2 National Institute for Research and Development in Electrochemistry and Condensed Matter, Plautius Andronescu Street 1, 300224 Timișoara, Romania; mihaelabirdeanu@gmail.com; 3 Department of Pharmacology-Pharmacotherapy, Faculty of Pharmacy, “Victor Babeş” University of Medicine and Pharmacy Timișoara, Eftimie Murgu Sq. No. 2, 300041 Timişoara, Romania; diana.utu@umft.ro; 4 Department of Preventive, Community Dentistry and Oral Health, Center for Advanced Technologies in Dental Prosthodontics, Faculty of Dental Medicine, “Victor Babeș” University of Medicine and Pharmacy Timișoara, Eftimie Murgu Sq. No. 2, 300041 Timișoara, Romania; matichescu.anamaria@umft.ro

**Keywords:** CAD/CAM provisional materials, in vitro wear tests, wear parameters, surface topography, clinical performance

## Abstract

In order to provide clinically significant evidence on the long-term functional performance of CAD/CAM provisional materials, especially 3D-printed and milled resins, accurate tribologically in vitro wear tests that integrate wear parameters and surface topography analysis are necessary. The goal of the study was to assess the wear resistance of several CAM-obtained dental crown materials and the relationship between wear and the manufacturing process, distinctive postprocessing, microhardness, microroughness, and surface topography. A standardized ball-on-flat tribological protocol was applied to (*n* = 70) CAD/CAM-fabricated PMMA specimens (four 3D-printed groups with distinct post-processing protocols (Optiprint) and three milled materials (TelioCAD, Shaded PMMA, Copra Temp Symphony)) to quantify wear parameters micro- and nanoroughness (Ra, Rz, Sa, Sy), and Vickers microhardness, followed by comprehensive statistical analysis (*t*-tests, Pearson correlations) to elucidate material- and process-dependent differences in wear behaviour. Nanoroughness was carried using atomic force microscopy evaluation. Wear testing showed that most materials, particularly the 3D-printed groups, developed limited wear, whereas the milled materials evolved toward groove-dominated wear topographies. Wear statistics showed that the printed resins consistently had an advantage, meaning that the degree and rate of wear are significantly influenced by the manufacturing process. Hardness has a central role in governing the wear performance of interim resin materials, while nanoroughness acts as a secondary factor. Optimised post-processing of printed materials, particularly a prolonged post-curing period, yields a beneficial combination of low wear and specific topography, thereby providing a significant clinical advantage.

## 1. Introduction

The growing interest in computer-aided design (CAD) and computer-aided manufacturing (CAM) technologies in dentistry has been accompanied by the introduction of a wide range of novel materials for provisional restorations. Clinical indications include the management of advanced erosive tooth wear with loss of vertical dimension and the use of staged approaches to future esthetic rehabilitation.

The wear resistance of these materials is a critical determinant of clinical decision-making and long-term maintenance of the restored vertical dimension. Yet, data on their long-term wear performance remain limited. In vitro investigations provide a controlled framework for characterising wear behaviour under standardised conditions. A comparative assessment of the wear behaviour of 3D-printed and milled CAM-fabricated provisional restorations may therefore yield clinically relevant information regarding the performance and indications of these newly developed materials [1].

Over the past years, research on wear of dental materials has progressed from simple hardness measurements to more sophisticated biomechanical and tribological investigations [2]. Increasing emphasis is now placed on characterising wear couples (i.e., the interaction of antagonist pairs), elucidating the influence of lubrication and the complex oral environment, and on the engineering of advanced restorative materials that exhibit improved durability, while minimising antagonistic tooth wear [3].

Wear is defined as the continuous, progressive loss of surface material, resulting from mechanical interaction between two bodies in relative motion. This biomechanical phenomenon can be experimentally simulated, with particular emphasis on attrition, by employing two-body wear configurations. In vitro investigations provide a relatively simple and time-efficient means to assess the wear behaviour of provisional restorative materials. Selecting clinically relevant wear parameters that can be translated into in vitro models is critical for generating meaningful data. Quantification commonly includes measurement of the worn area and the associated volumetric material loss, while the topography of the wear scar is frequently characterised using surface profilometry [4]. When it comes to dental materials, this test configuration has clear advantages over more traditional methods like pin-on-disk setups or chewing simulators because it produces near-circular wear scars that replicate the key topographical characteristics of clinically observed wear facets and allows relatively simple quantification of wear rate.

Attrition refers to mechanical damage of dental hard tissues caused by direct tooth-to-tooth contact and is, in principle, classified as a two-body abrasive process. Studies have demonstrated that lateral mandibular movements (e.g., grinding) produce significantly greater tooth wear than purely vertical contacts [2,3]. Bruxism is the most severe and clinically relevant contributor to common tooth wear, with occlusal forces reported to reach approximately six times those generated during normal mastication [4,5].

Surface topography exerts a crucial influence on the functional properties of materials, including friction, adhesion, optical behaviour, and interaction with the surrounding environment [6]. Consequently, precise quantitative and qualitative characterisation of surface texture is indispensable for evaluating both the appearance and functional performance of dental surfaces. In dentistry, a variety of laboratory protocols are employed to assess the wear behaviour of materials, each based on distinct tribological concepts and yielding different outcome measures such as volumetric loss, surface roughness, or topographic alterations [7,8]. Careful selection and interpretation of these methods are essential for obtaining clinically meaningful information on the wear resistance of contemporary restorative CAD/CAM materials [9].

ISO/TS 14569-2:2001 [10] establishes a standardised laboratory framework for assessing the wear resistance of dental materials under two- and three-body contact conditions, which replicate occlusal interactions between opposing teeth. This technical specification led to a set of standardised testing procedures under precisely controlled parameters of load, speed, environment (water or abrasive slurry), and test duration. Wear is quantitatively determined through measurements of mass loss and/or profilometric assessment of volume and height variations relative to designated reference materials [2]. The biomechanical wear tests are still not standardised with respect to antagonist type, force, frequency, number of cycles, environmental conditions, and surface polishing before environmental exposure and before testing. Teeth wear is primarily caused by tooth-to-tooth contact resulting from bruxism, clenching, and parafunctional pressures, which occur at an average rate of 8.1 cycles per hour. This is equivalent to 8.1 cycles × 24 h × 365 days = 70,956 cycles per year, or 5913 cycles per month [4].

Alternative testing methodologies vary in several parameters, including the applied load, the number and frequency of cycles, and the testing environment. Although wear testing is not mandatory for dental materials prior to market release, when such evaluations are performed, the results should be supported by appropriate laboratory investigations. Clinically, the significance of wear is primarily related to its detrimental impact on the functional integrity of the restoration and/or the aesthetic appearance [3].

Surface topography influences not only the aesthetic appearance of a restoration but also its texture. Furthermore, surface roughness plays a significant role in determining the amount of material wear, making the control of surface topography particularly important. ISO 25178-2:2021 [10] is the first international standard to characterise three-dimensional surface texture, providing a coherent framework for the specification, measurement, and verification of 3D surface topography. The standard introduces harmonised terminology, symbols, and parameter sets for surface analysis. It addresses the measurement and interpretation of surface nanoroughness. Among the height parameters defined, the arithmetical mean height (Sa) is the arithmetic mean of the absolute deviations of the surface height values from the mean plane, while the maximum height (Sy) is the vertical distance between the highest and lowest points on the surface [11]. AFM is a cantilever-based method that examines surfaces at resolutions below the optical iffraction limit using a sharp tip. Additionally, it is an efficient device for measurements and nano-mechanical exploration [12]. Profilometric measurements were conducted in six randomly selected regions within the central and peripheral wear facets and on the polished surfaces.

Surface roughness at the micron scale, typically expressed by parameters such as Ra (arithmetic mean roughness) and Rz (average maximum height), is a critical characteristic of interim dental restorations. Increased surface roughness has been shown to substantially promote biofilm accumulation. Additionally, a roughened provisional restoration may contribute to excessive wear of the opposing natural dentition [11]. Ra is the most commonly employed parameter for quantifying surface roughness. It represents the arithmetic mean of the absolute deviations in profile height within a defined assessment length, providing an overall measure of average surface irregularity. However, Ra may not adequately capture isolated extreme peaks or valleys. In contrast, Rz denotes the mean value of the absolute heights of the five highest peaks and the depths of the five deepest valleys across the same assessment length. This parameter is more sensitive to pronounced surface irregularities and reflects the maximum height variation in the surface profile. Consequently, even when the Ra value is low, a high Rz value indicates the presence of deep scratches or pronounced asperities that may serve as potential initiation sites for crack propagation.

CAD/CAM milled materials are fabricated from pre-polymerised, highly cross-linked PMMA blocks processed industrially under elevated pressure and temperature, resulting in a densely polymerised material with reduced porosity and fewer internal defects. Following milling, they present a relatively smooth surface, which typically requires only minimal finishing to remove machining marks and can be brought to a high-gloss polish relatively easily. By contrast, the surface roughness of 3D-printed resins is strongly influenced by printing parameters and the quality of post-processing and polishing procedures [11].

CAD/CAM-milled provisional resins are typically fabricated from pre-polymerised material. This microstructural homogeneity is associated with superior mechanical properties and reduced volumetric wear compared with conventionally polymerised resins used for interim restorations. These materials are frequently reported to exhibit favourable antagonist-friendly behaviour, as their high surface hardness can be combined with excellent polishability, allowing a very smooth surface that minimises abrasive effects on opposing dentition [13]. Consequently, CAD/CAM milled PMMA has often been regarded as the reference material for provisional restorations and is routinely used as a benchmark in comparative studies evaluating newer 3D-printed resins and other digitally manufactured interim materials [14].

3D-printed resins produced by group. Vat-polymerisation technologies such as SLA and DLP constitute a rapidly evolving class of materials in which restorations are fabricated layer-by-layer and subsequently post-cured with UV light. Early generations of these resins exhibited relatively poor wear resistance, but recent formulations have shown substantial improvements, although their wear behaviour often remains inferior to that of CAD/CAM-milled materials in many comparative investigations [15]. The inherent layer-wise architecture and the potential for partial monomer conversion can result in rougher surfaces and structurally weaker interfaces, which could serve as wear and micro-damage initiation sites [16].

Several in vitro studies indicate that certain contemporary 3D-printed provisional resins can achieve wear resistance and antagonist wear patterns comparable to those of milled PMMA-based materials, particularly when optimised printing orientations and finishing protocols are applied. Research activity in this field is intense, and new 3D-printing materials specifically designed for long-term provisional use are progressively narrowing the performance gap with subtractive-manufactured resins. Post-processing conditions, especially post-curing time and temperature, play a critical role in enhancing the degree of conversion, mechanical strength, and wear resistance, emphasising the need for rigorously controlled protocols [16,17].

Some recent investigations also suggest that, under carefully selected parameters (such as favourable build orientation and homogenised microstructure), 3D-printed materials can demonstrate wear resistance that equals or even surpasses that of certain milled or conventionally fabricated resins [18]. Crosslinking modifications alone, however, have not consistently produced significant gains in wear behaviour, indicating that overall formulation and processing conditions are more decisive. Overall, digital fabrication methods, both 3D printing and CAD/CAM milling, tend to yield superior wear resistance compared with traditional provisional materials, making them attractive options for contemporary interim restorations [15,16,19]. The chemical composition of the printed and milled samples is similar; these are PMMA-based materials. The printed resin consists of highly filled methacrylate resin, layer-wise structure, post-cured and the milled resins are high-conversion PMMA disc with a compact, low-porosity matrix. These types of resins are indicated for long term interim prosthetic restorations.

The aim of the study was to evaluate the wear resistance of different CAM-obtained dental crown materials, including vertical and volumetric loss, and to assess the correlation between wear and the manufacturing method, postprocessing, microhardness, microroughness, and surface topography. The following null hypotheses were formulated:

**H01:**  *There is no statistically significant difference in wear behaviour among the tested CAM-obtained materials, regardless of manufacturing method.*

**H02:**  *The manufacturing method (milling vs. 3D printing) has no statistically significant effect on surface roughness or surface topography of the crown materials, related to wear.*

**H03:**  *There is no statistically significant correlation between microhardness and wear behaviour of the tested materials.*

**H04:**  *Different post-processing protocols of the printed materials do not significantly influence the wear behaviour of the tested materials.*

## 2. Materials and Methods

### 2.1. Specimen Preparation

A 3D-printed crown material with various post-processing settings and three types of CAM-milled materials were employed in the study (Table 1). Glycerin was applied only to the fourth group of resin samples as a part of the post-processing step. G* Power software v3.0.10 was used to calculate the sample size. Assuming a normal distribution and the effect size (0.49), the necessary sample size was computed for α = 0.05 and a power of 0.90. Eight samples per group are needed for statistical significance, according to the computation. A larger sample increases the credibility of the results and more accurately reflects the genuine variety of materials and structures [20]. Consequently, this study used 70 specimens (n = 10 per material group).

Fusion software (Autodesk Inc., San Francisco, USA) was used to design the rectangular plates (10 × 10 × 1 mm), which were then produced using a DLP 3D printer (Asiga Max UV, Asiga, Sydney, Australia) at a build angle of 0° and a layer thickness of 50 μm. After five minutes of ultrasonic rinsing in an isopropyl alcohol solution, the plates were post-cured differently for each group using a BB Midi Plus post-curing equipment (MECCATRONICORE, Pergine Valsugana, Italy) with triple-frequency technology (365, 405, 480 nm). The blocks were sectioned to the same size for the subtractive samples. Silicon carbide paper was used to gradually polish the specimens to #2000 grit. All specimens were stored in distilled water at room temperature prior to testing.

The producer of the printed resin recommends using room temperature during the post-curing process in the post-polymerisation step. The post-processing characteristics were chosen for the first group at 22 degrees Celsius and for 7 min.

Although Dentona’s official protocol for Optiprint Lumina emphasises light intensity (Otoflash G171) instead of specifying a heat setting, independent scientific literature consistently identifies 60 °C as the “gold standard” for post-processing dental resins. A temperature of 60 degrees was selected for the second group.

### 2.2. Wear Tests

**Figure 1:**
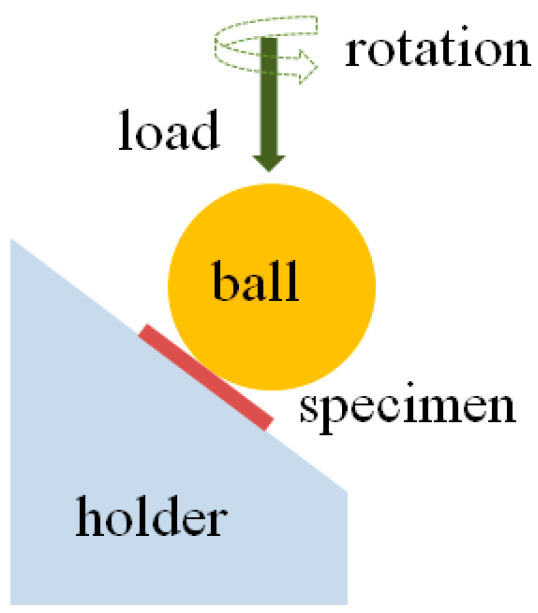
Illustration of the tribotester, with a mounted specimen.

Material specimens were mounted in a specially designed tribometer testing machine [21]. Figure 1 illustrates the rotating ball-on-flat tribotester.

On the specimen plates, a 6.35 mm-radius zirconia sphere is loaded normally with 30 N and rotated at 30 rpm. The angle formed by the line from the sphere centre to the specimen contact and the rotation axis is 37° as illustrated in Figure 1.

Flat specimens produce distinct, nearly circular wear scars from which wear parameters can be assessed.

PMMA flat specimens were used in opposition to spherical zirconia ball antagonists. The hardness of the zirconia balls (mean Vickers hardness: 1325.6 GPa) was measured.

The specimens were directly impacted by the antagonist at 30 rpm, with a mean load of 30 N. To minimise thermal effects and prevent additional heat-induced damage to the specimens, this load–frequency combination was chosen to approximate physiological masticatory conditions [21].

To simulate saliva, a 33% glycerin solution was employed as a lubricant. The tests were conducted at room temperature [22]. Glycerin was added on the surface of all tested samples.

At cumulative test duration t, the sliding distance L across each scar was calculated as in Equation (1)

(1) L = 2πRftsinθ

where θ = 37° is the angle between the line from the sphere centre to the specimen contact and the rotation axis.

**Figure 2:**
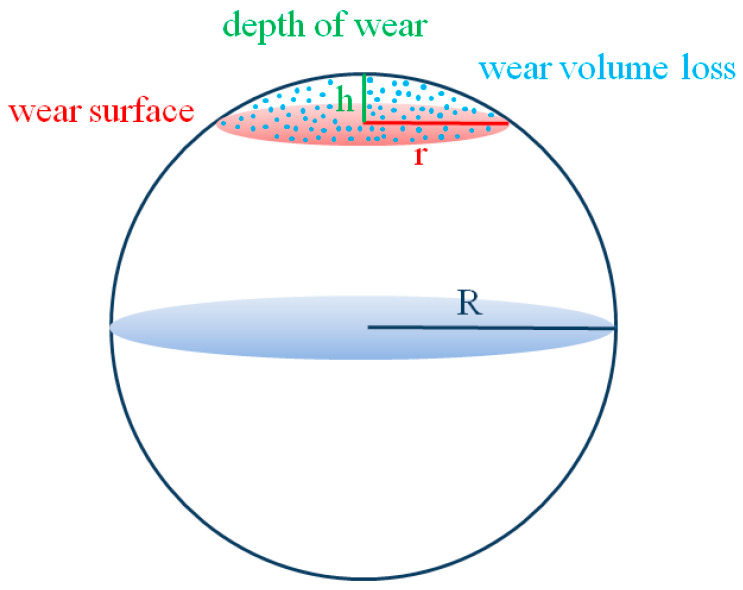
Schematic diagram for the representation of the depth of wear, wear surface, and volumetric wear.

Both parallel and perpendicular to the sliding direction, optical measurements of the scar radii r were made, and average values were computed. ImageJ (version 1.46, Java) was used to measure the radius of the counter body and the wear scar. Figure 2 images were inserted into the program, and the previous data was calculated. In this study, each specimen underwent 5913 cycles, equivalent to one month of use.

After the wear simulation experiments, the specimens were ultrasonically cleaned in distilled water for 3 min before examination.

**Figure 3:**
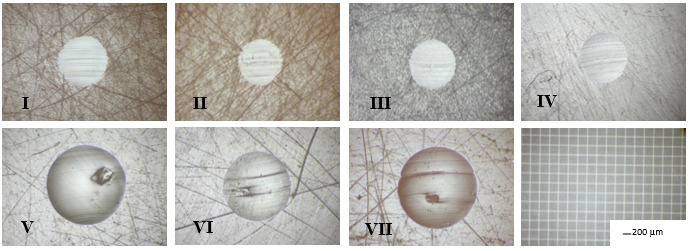
Optical microscopic images of wear scars.

The form of the wear scar was examined using low-magnification micrographs. The plates with near-circular wear scars were examined using optical microscopy at a 4× magnification (Leica DM 100; Mannheim, Germany). Data regarding wear surface area and volumetric wear were evaluated [23] (Figure 3).

Applying the simple conformational relation, scar volumes V have been calculated [21].

(2) V = πr^4^/4R

Using formula the greatest depth of wear h (μm) was found, and mean values were computed [24].

(3) h = R − (R^2^ − r^2^)^1/2^

where R is the radius of the counter ball, and r is the radius of the wear scar.

The Formula (4) was used to determine the wear rate (WR), which is the rate of material loss [25].

(4) WR (μm/1000 cycles) = Maximum depth of wear (μm) × 10^3^/number of cycles

According to Archard’s law (20), the specific wear rate SWR was calculated using the formula [12]

(5) SWR = V/PL [×10^−3^ mm^3^/Nm]

where V is the volume loss, P is the applied normal load (force), and L is the total sliding distance.

Lower specific wear rates indicate better wear resistance.

#### 2.2.1. Assessments of Surface Roughness

The surface roughness of the specimens was examined using a Surftest SJ201 surface profilometer (Mitutoyo, Kawasaki, Japan). Eight directions were used to assess arithmetic average roughness (Ra) and maximum absolute vertical roughness (Rz) on polished and worn surfaces, and all data were recorded. For every surface, the average of the measurements was determined. A force of 0.7 mN was applied, and the sampling length was 0.8 mm.

#### 2.2.2. Topographic Characterisation of Nanosurfaces Using Atomic Force Microscopy (AFM)

A surface profilometer was used to analyse the surface shape and wear pattern generated following tribological testing. Two-dimensional and three-dimensional surface profiles, as well as the average surface roughness data, were recorded. An atomic force microscope in contact mode, the Nanosurf Easy Scan 2 Advanced Research (NanosurfAG, Liestal, Switzerland), was used to investigate the samples. The heights Sy (nm) and the average nanoroughness Sa (nm) were measured. The AFM analyses provided a 2.2 µm × 2.2 µm three-dimensional image of the sample surfaces.

#### 2.2.3. Measurements of Microhardness

A microhardness indenter DM 8/DM 2 (Yang Yi Technology Co., Ltd., Tainan City 70960, Taiwan) was used. Applying a diamond pyramidal indenter with a dwell period of 10 s and a load of 300 g, five indents were produced on each specimen at specific locations using a digital camera. The dimensions of the indentation were measured under a microscope (40× magnification) after the indenter was lifted. Each surface was measured five times, and mean values were computed.

The Formula (6) was used to determine Vickers hardness (VH).

(6) VH = (1.8544P)/D

where P is the load, and D is the average diameter.

### 2.3. Statistical Analyses

Means ± standard deviations were used to express all calculated and measured data. To evaluate differences across materials, an unpaired Student’s *t*-test was used to examine data for each stage. The values before and after wear were compared using a paired Student’s *t*-test. The dependency between variables recorded across phases was assessed using regression and Pearson correlation; 0 to 0.2 (very weak), 0.2 to 0.4 (weak), 0.4 to 0.6 (moderate), 0.6 to 0.8 (strong), and 0.8 to 1.0 (very strong) were all related to the significance of the Pearson coefficient (r). A value of 0.05 was chosen as the level of significance.

## 3. Results

### 3.1. Wear Tests

Images of wear scars in the tested materials, at the end of each test (t = 200 min, L = 144 mm) are shown in Figure 3. All of the images were taken and are shown at the same magnification. Although the tested materials and the rotating zirconia sphere were initially smooth, scratch tracks are evident across the scar widths, indicating the presence of developing third-body particles in the test medium. The third body particles result after the two-body test, as residuals form the samples and the glycerin environment.

**Figure 4:**
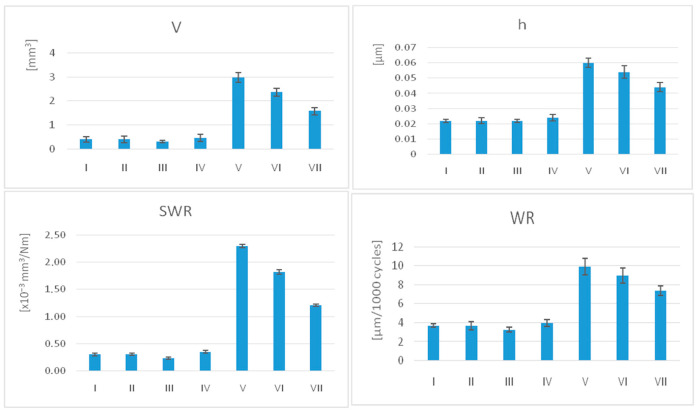
The graphical representation of V, h, SWR, and WR of the samples, for all materials.

The average values for volumetric loss (V), height of the vertical loss (h), specific wear rate (SWR), and wear rate (WR) with the standard deviation (SD) are summarised in Table 2, Table 3 and Table 4 and shown in Figure 4.

The lowest average values of SWR and WR were recorded for III (SWR = 0.071 ± 0.255, WR = 3.252 ± 0.31), while V experienced the most severe wear (SWR = 0.69 ± 1.196, WR = 9.926 ± 0.34), followed by VI (SWR = 0.548 ± 1.174, WR = 8.995 ± 0.48).

For SWR, WR, V, and h, printed materials show significantly lower values than milled samples after unpaired *t*-tests (*p* < 0.001). As well as significant variations within these groups associated with the same manufacturing technology, these groups were assessed. Related to the post-processing methods for 3D printed samples, group III recorded significantly lower wear rates than groups I and IV.

### 3.2. Assessments of Surface Roughness

**Figure 5:**
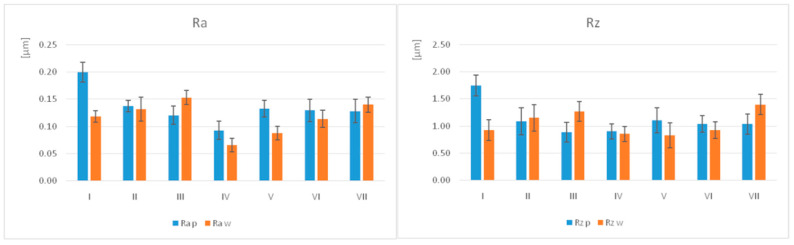
Graphical representation of surface roughness Ra and Rz for all groups (p = polished, w = worn).

The average values of surface roughness Ra (µm) and Rz (µm), with the standard deviation (SD) for polished (*p*) and worn (w) samples, are summarised in Table 5 and illustrated in Figure 5.

For the polished samples, the highest Ra value was recorded for I (0.20 ± 0.02) and the lowest for IV (0.09 ± 0.02), whereas for the other materials, the values were almost similar. For the samples subjected to the wear test, the highest value was for III (0.15 ± 0.01) and the lowest for IV (0.07 ± 0.01). Regarding the Rz values on the polished surfaces, the highest values were measured for I (1.75 ± 0.26), while the lowest were for III and IV (0.89 ± 0.01). On the worn surfaces, the highest value was recorded for VII (1.40 ± 0.02) and the lowest for V (0.83 ± 0.23).

The surface roughness decreased after the wear test for most materials, more for I, followed by V and slightly increased for III and VII.

Regarding microroughness, there are no significant differences in processing technology. Within groups, there are significant differences in the printed group, both polished and worn, and for the milled group, only for the worn (Table 6).

### 3.3. Topographic Characterisation of Nanosurfaces Using Atomic Force Microscopy (AFM)

**Figure 6:**
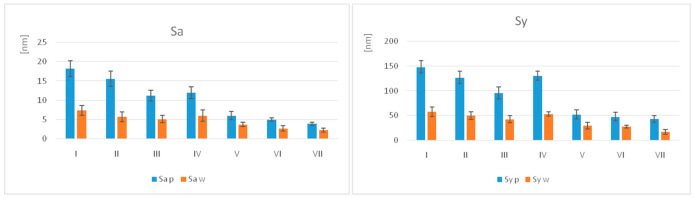
Graphical representation of surface nanoroughness Sa and Sy for all groups. Sa—arithmetical mean height, Sy—maximum height, p = polished, w = worn samples.

The values of the parameters Sa (nm) and Sy (nm), along with their standard deviations (SD), are summarised in Table 7 and shown graphically in Figure 6.

For the polished samples, the highest Sa value was recorded for I (18.20 ± 2.53) and the lowest for VII (3.90 ± 0.43). For the samples subjected to the wear test, the highest Sa value was for I (7.35 ± 1.25) and the lowest for VII (2.27 ± 0.92). Regarding the Sq values on the polished surfaces, the highest values were recorded for I (22.93 ± 1.26), and the lowest were for VII (5.23 ± 0.43). On the worn surfaces, the highest mean value was registered for I (9.13 ± 1.25) and the lowest for VII (2.78 ± 0.50).

The nanoroughness decreased for all materials, with the greatest decrease in I, followed by II.

The statistical analysis (unpaired *t*-test, α = 0.05) of the polished samples showed significant differences (*p* ≤ 0.05) in Sa and Sy between almost all groups, except for I–II, II–III, IV–VII, and V–VI. Among the groups of materials subjected to the wear test, significant differences were observed, except for I–II, III–IV, II–III, IV–VII, and VII–VI.

The statistical analysis (paired *t*-test, α = 0.05) reported significant differences (*p* ≤ 0.05) in nanoroughness between the polished and worn samples of the same material, except for IV and VII, for Sa (Table 8).

In terms of nanoroughness, unpaired *t*-tests revealed significant differences of the samples related to the processing technology.

Within groups, nanoroughness decreases significantly after wear.

All four 3D-printed interim resin materials exhibit a clear reduction in surface topography amplitude during in vitro wear, as shown in atomic force microscopy images of the printed samples. This suggests that the occlusal contact area has been effectively smoothed and that visible asperities have been eliminated.

All materials exhibit a quite rough texture prior to wear, with deep valleys and sharp peaks across the 2.2 × 2.2 µm scan area, indicative of layer-by-layer printing and insufficient post-processing smoothing.

**Figure 7:**
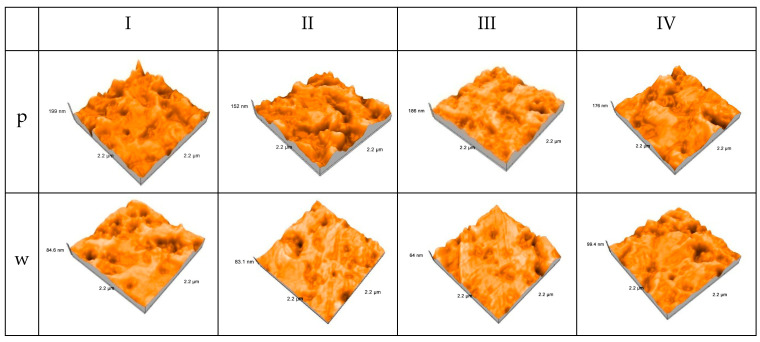
Three-dimensional AFM surface topography of 3D-printed interim resin materials before and after in vitro wear (p—polished, w—worn).

The same materials exhibit smaller depressions, more softened peaks, and a generally more uniform topography after wear, indicating that sliding contact has partially levelled the surface and selectively eliminated high asperities (Figure 7).

Group I appears to have the largest peak-to-valley height, which remains relatively high after wear, suggesting a surface morphology that is less easily smoothed and more wear-resistant.

Group II and III exhibit a significant reduction in peak height and a more uniform texture after wear, indicating a matrix that undergoes little abrasion and plastic deformation, resulting in a smoother worn track.

Group IV exhibits the least qualitative change, retaining wear-induced residual imperfections. This could be due to a more durable resin matrix that restricts topographical flattening under the tested conditions.

**Figure 8:**
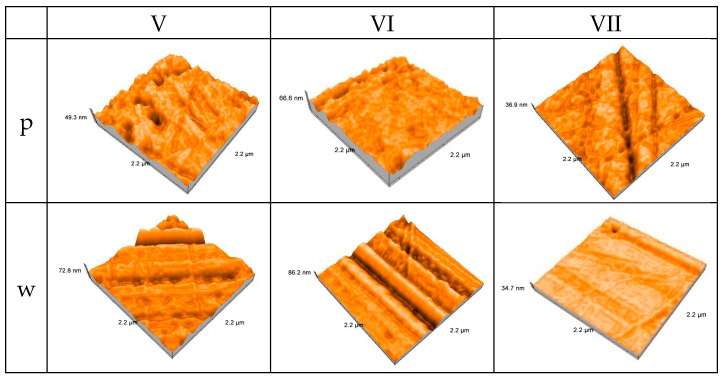
Three-dimensional AFM surface topography of milled interim resin materials before and after in vitro wear (p—polished, w—worn).

All specimens exhibit directionally structured abrasion within the 2.2 × 2.2 µm scan zones, and the atomic force microscopy images indicate clear changes in surface topography for the three CAD/CAM-milled interim materials during in vitro wear (Figure 8).

The milled materials generally exhibit isotropic textures before wear, with a gently uneven surface and low peak-to-valley amplitudes corresponding to polishing marks.

Each material exhibits more pronounced linear features and plateau-like areas after wear, suggesting preferential material removal along the sliding direction and a surface that changes to an anisotropic, tribologically conditioned topography.

Group V wear results in wide grooves with little change in peak height, indicating a mostly moderate abrasion and strong resistance to deep ploughing.

Group VI exhibits higher peaks and sharper, deeper parallel grooves after wear, consistent with microploughing and suggesting a slightly more brittle or heterogeneous microstructure under the applied pressure.

With smoother, more continuous plateaus and less noticeable groove morphology, Group VII exhibits the lowest visible peak-to-valley amplitude post-wear, suggesting more uniform material removal and greater susceptibility toward surface smoothing under sliding.

The AFM data show how interim restorations respond to mechanical loading at the nano-topographical level, emphasising the need to combine qualitative AFM imaging with quantitative roughness and texture parameters when assessing wear behaviour of contemporary interim dental materials.

### 3.4. Measurements of Microhardness

**Figure 9:**
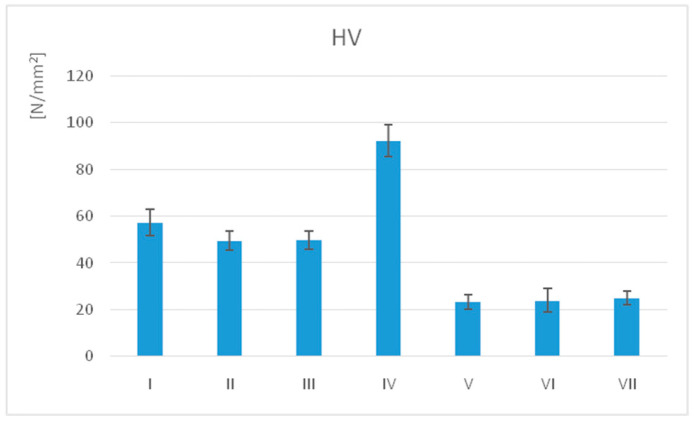
Graphical representation of the mean microhardness values ±SD on the surfaces of all materials.

The mean Vickers hardness (HV) values for the samples are presented in Table 9 and Table 10 and illustrated in Figure 9.

The highest HV value was recorded for IV (92.37 ± 6.90) and the lowest for V (23.23 ± 3.11).

The statistical analysis (unpaired *t*-test, α = 0.05) of the polished samples revealed significant differences (*p* ≤ 0.05) in surface microhardness across almost all groups, except II–III and V–VI.

Related to the processing technology, printed materials have significantly higher microhardness values than milled. Also, within-group differences were calculated.

While WR provides a simple output from a given experiment, SWR is known as an essential, standardised scientific metric. SWR transforms a condition-specific measurement into a fundamental material property, making it the critical parameter for ranking the long-term durability of dental restorative materials. SWR is a reliable indicator of true wear performance. The correlations between the SWR and the measured and calculated parameters are presented in Table 11.

These results indicate that hardness is the primary controlling factor in wear, whereas surface nanoroughness is a secondary factor. Increased bulk hardness is strongly associated with a decreased specific wear rate, meaning superior wear resistance. A higher HV indicates a greater resistance to plastic deformation and penetration. The negative correlation between Sa values and SWR may be attributed to the specific tribosystem. High nanoroughness (Sa) does not act as a stress concentrator for abrasion, but rather as a functional topography that optimises lubrication, mitigates contact pressure due to a larger surface area, and manages wear debris. This shifts the dominant wear mechanism toward mild wear, resulting in the observed correlation between higher Sa and lower SWR.

## 4. Discussion

Due to their lightweight nature, favourable aesthetic properties, ease of processing, polishing, and clinical handling, as well as their relatively low cost and stability in the oral environment, digitally processed PMMA materials are widely employed for interim dental restorations. Their physical, mechanical, and tribological characteristics enable them to withstand functional masticatory loads. In this context, a two-body tribological investigation was conducted on contemporary interim restorative materials in order to characterise their wear behaviour. Distinct surface morphologies were observed under specific loads, sliding speeds, and durations, revealing specific aspects of the worn surfaces [26].

In vitro wear testing of resin-based restorative materials primarily results in abrasive wear, which occurs when a spherical antagonist contacts the material surface, inducing a ploughing mechanism. This process leads to localised plastic deformation and subsequent material removal, forming a distinct wear track. The evaluation of short-term wear behaviour among these materials can be valuable for distinguishing their performance characteristics, particularly since such restorations are typically designed for temporary clinical application [27].

The test quantifies the relative wear susceptibility of temporary resin-based materials under hard contact [21]. The well-controlled test settings, which may not adequately reflect the complex nature of real-life oral motion, may be one of the study’s limitations [28].

Previous studies show that, despite their different physical structures, milled polymer materials and novel printed polymer resin restorative materials may provide comparable wear resistance. Antagonists were less worn and damaged by printed substrates [29]. According to additional research, 3D-printed materials exhibit the highest wear resistance [30], whereas the wear resistance of 3D-printed resin materials falls within a range similar to that of milled or conventionally manufactured resin materials [1,18]. The wear volume loss of the 3D-printed and milled resins was found to be lower than that of the conventional resin [31].

Based on other research, occlusal functional loading is going to degrade the mechanical characteristics of resin-based dental materials in the oral cavity, leading to wear and increased surface roughness [32]. It is anticipated that normal dentition undergoes wear of 15–29 μm annually [33]. Maintaining wear rates comparable to those of natural dentition is challenging for restorative materials. As a result, wear testing is essential for assessing the materials topography and wear resistance. Additionally, it is very difficult to measure wear rates accurately below 50 μm [34]. Research that thoroughly evaluates various provisional materials, taking into account their wear resistance over a predetermined period, is therefore required. To improve understanding of the dental resin-based materials available for temporary restorations, analyses of surface topography, vertical loss (2D maximum depth of the material), and volume loss (3D total volume loss across the whole area) would be of interest.

Indirect wear measures were defined as area and depth. Because wear is a dynamic process, occlusal variables that change over time, as wear progresses, define both the wear area and wear depth. As a result, it is crucial to integrate the evaluations of wear depth and wear area as suitable wear quantities. It has been suggested that applied force, time, sliding speed, and environmental conditions contribute to the amount of material removed during the interaction of opposing surfaces. It was recommended that volumetric wear be used as the preferred criterion for describing the in vitro wear of dental restorative materials, that wear volume is a material feature and thereby treating wear volume as a material property [35]. In relation to earlier research, the wear behaviour was explored in the current study considering the masticating force of 30 N and a rotation speed of 30 rpm [25,36].

The overall material integrity can be determined from the volumetric loss data. According to earlier research, 3D wear measures are better than 2D ones since they offer a more comprehensive picture of wear loss [28,35]. Volumetric loss among materials may not reflect a proportional amount of vertical loss, according to the results of volumetric and vertical tests. Vertical loss is restricted to linear measures, whereas volumetric loss reflects total surface wear. It would be easier to fully understand the wear dynamics if both vertical and volumetric loss were used [33].

Volume and vertical loss were highly associated, regardless of the measurement technique. Since the occlusal contact sites stabilise the vertical distance between the maxilla and mandible, the maximal height loss is the more clinically significant indicator in dentistry [37]. Evaluating the wear volume frequently consists of briefly examining the wear surfaces [27,28].

In this study, the wear behaviour of interim dental resin materials was evaluated in terms of volumetric loss (V), vertical loss height (h), specific wear rate (SWR), and wear rate (WR). The concurrent assessment of them is required to capture not only the magnitude and spatial distribution of material loss but also the normalised and time-dependent aspects of wear, thereby enabling robust comparison and clinically relevant interpretation of the wear behaviour of interim dental resins [38]. Volumetric loss (V) quantifies the total amount of material removed and is essential for assessing the overall severity of wear and comparing bulk material loss between groups. Vertical loss height (h) reflects the depth of the wear track, providing information on local surface damage, potential compromise of occlusal morphology, and clinical fit. Specific wear rate (SWR) quantifies wear as a function of load and sliding distance, enabling meaningful comparisons across test conditions, materials, and studies by accounting for mechanical and kinematic variables. Wear rate (WR) quantifies wear progression over time or cycles, providing insight into degradation kinetics and enabling prediction of long-term performance from short-term tests.

The four wear parameters used in the research provide complementary information on how the printed (I–IV) and milled (V–VII) interim materials behave under in vitro wear conditions, and together they show a clear advantage for the printed resins. Across all seven materials, V, h, SWR, and WR show a consistent pattern: higher values cluster in the milled group and lower values in the printed group, demonstrating that the manufacturing method strongly influences both the magnitude and rate of wear.

**H01:**  *There is no statistically significant difference in wear behaviour among the tested CAM obtained materials, related to manufacturing method was rejected.*

Related to the surface topography, previous studies demonstrated that after simulated chewing, the 3D printed group’s surface was smoother than the milled and conventional PMMA group’s. Material wear results in a rougher surface and encourages increased accumulation of plaque on the worn surfaces of both traditionally and digitally manufactured interim restorative materials [31].

A rough surface accelerates the wear of both the restorative material and its antagonist. Therefore, achieving an optimal surface finish through appropriate polishing is crucial for all restorative systems, particularly for conventional and 3D-printed materials. Recent studies consistently highlight that surface finishing and polishing play a role as significant as the material composition itself in determining wear performance. The wear resistance of interim restorative materials is strongly influenced by their inherent polishability and the finishing protocol employed. Continuous research efforts focus on evaluating the effectiveness of newly developed universal polishing systems in optimising surface characteristics of these innovative materials. Additionally, the interaction between two restorative materials (e.g., a zirconia crown opposing an interim crown) represents a key factor affecting clinical longevity and functional stability. The predominant wear mechanism is matrix degradation, which is strongly influenced by surface polishing quality [39,40].

The wear of resins may be influenced by their physical characteristics. The wear pattern in direct contact between resin and antagonist is mostly a mix of microfatigue and attrition/abrasive wear. The wear rate of resins is determined by the friction coefficient and surface roughness [3]. Differences between groups I and IV of the printed resin samples, which underwent the same post-processing protocol except that the IV group included glycerin, can be explained by glycerin’s ability to prevent the formation of a weak post-polymerisation layer, thereby significantly increasing surface hardness. Oxygen inhibition had a positive effect on surface hardness and nanoroughness; however, it significantly decreased wear resistance under the tested tribological conditions. Mechanical characteristics and surface quality were significantly impacted by finishing and polishing procedures. After completion, applying a glaze may increase strength and decrease roughness. Significant differences were observed between Groups I and IV after statistical analysis, which can also be explained by the addition of the glycerin layer in the post-processing steps [41].

According to available data, 3D-printed resins typically exhibit higher initial roughness, requiring careful post-processing to achieve equivalent surface quality, whereas digitally milled materials consistently exhibit the lowest and most uniform Ra and Rz values [11].

In everyday use, interim resin restorations are frequently adjusted, and finishing and polishing procedures are crucial. While polishing reduces roughness and gives the surface a glossy, enamel-like look, finishing eliminates adjustment scratches and produces a smooth surface. Colour, biofilm retention, and gingival irritation can all be impacted by poor finishing and polishing, which can result in secondary cavities and restoration failure. The mechanical qualities and roughness can be affected by the polishing and finishing process. Maintaining the functional and aesthetic needs of replaced teeth depends on the surface characteristics of restorative materials, which are clinically important components. The lifespan of restorations is also significantly influenced by surface characteristics. To prevent bacterial retention and biofilm formation, a clinically significant mean roughness (Ra) threshold of about 0.2 μm has been proposed [41].

Microroughness Ra values show that, at the micrometric scale, all materials remain within the clinically acceptable range, with initial Ra values on polished samples roughly between 0.09–0.20 µm and post-wear Ra values between about 0.06–0.15 µm.

In this study, for printed resins, AFM images show a marked reduction in peak-to-valley amplitude, which suggests that occlusal loading can decrease local roughness and potentially reduce plaque retention and antagonist abrasion, but the extent of smoothing differs between materials, indicating distinct interactions and wear mechanisms. For milled polymers, the directional grooves indicates a transition to functionally adapted, tribologically conditioned surfaces, where friction and wear response become direction dependent; the depth and definition of these grooves vary among materials, suggesting different resistance to loading.

A systematic reduction in the arithmetic nanoroughness Sa after in vitro wear was observed for all four additive and three subtractive interim restorative materials. Sa values are higher for the additive materials, whereas the subtractive materials start with lower initial Sa, indicating a finer initial topography for the subtractive technology. After wear, Sa decreases in all groups, with a relatively larger reduction in the additive materials, indicating that tribological loading efficiently smoothes the pronounced asperities generated by printing, whereas the milled materials show a more moderate reduction in roughness. For the four additive materials, the large Sap–Saw difference indicates wear dominated by matrix abrasion and plastic deformation, transforming an initially rough microtopography into a more levelled nanoscale surface. In the subtractive materials, the already low initial Sa means that Sap–Saw changes are smaller.

**H02:**  *The manufacturing method (milling vs. 3D printing) has no statistically significant effect on surface roughness or surface topography of the crown materials, related to wear is partially accepted, only for microroughness.*

Microhardness is an important physical characteristic to explain wear, even if it cannot predict wear performance on its own [22]. Because the hardness of these materials increases the vulnerability of opposing restorations, the development of monolithic zirconia-based dental restorations brought wear into attention. The wear of zirconia against other restorative dental materials has not been extensively investigated [42,43,44]. The current study used a well-established testing technique using a hard zirconia spherical contact to assess the relative wear susceptibilities of resin-based dental materials. The abrasiveness of a substance is actually determined through microhardness measurement. According to several studies, materials with strong microhardness can result in high antagonist wear [45,46].

Across all studied materials, higher Vickers microhardness tends to be associated with lower wear rates, confirming the central role of hardness in governing the wear performance of interim resin materials.

The combination of high HV with low wear rates suggests a microstructure that resists plastic deformation and maintains a stable surface under loading, supporting its superior tribological and clinical profile among the printed materials.

**H03:**  *There is no statistically significant correlation between microhardness and the wear behaviour of the tested materials is rejected.*

Post-processing represents a critical yet frequently underestimated phase in the fabrication of interim dental restorations, as it has a decisive influence on biocompatibility, mechanical performance, surface characteristics, and aesthetic outcome. The required post-processing protocols differ substantially between subtractive CAD/CAM-milled polymers and additively manufactured (3D-printed) resins, particularly with respect to cleaning, post-polymerisation, and polishing procedures. Adequate post-processing reduces residual monomer and improves the degree of conversion, thereby enhancing biocompatibility and minimising cytotoxic and inflammatory responses. At the same time, it improves flexural strength, hardness, and dimensional accuracy, while enabling a smooth, highly polished surface that limits plaque accumulation and antagonistic wear. Conversely, suboptimal post-processing has been identified as a major contributor to clinical complications, including fracture, discolouration, surface degradation, marginal inaccuracy, and soft-tissue irritation, ultimately compromising the longevity of interim restorations [47,48].

Post-processing is generally more critical for additive (3D-printed) technologies than for milled materials. This is mainly because light-cured printed resins inherently present a superficial layer of uncured or partially cured material and are highly sensitive to washing and postcuring protocols, which directly affect residual monomer content, biocompatibility, and mechanical properties [49]. Fast acrylate monomer polymerisation and brief radiation exposures are crucial components of photopolymerization-based printing. Compared with traditional photopolymerized composite resins, printing resins contain higher levels of photoinitiators (3–5%) to increase polymerisation reactivity per short cycle. After the reaction is complete, unreacted initiators and monomers remain. Consequently, post-processing with more radiation is necessary to finish the polymerization process. Manufacturers advise using polymerisation equipment with a particular range of wavelengths that are suitable for the printing resins for this post-polymerisation process, which affects the colour, translucency, microhardness, and flexural strength of 3D printed interim resin materials [50].

Several recent studies have assessed the effects of post-processing variables on mechanical properties, including rinse duration, rinsing solution, polymerisation time, polymerisation equipment, and artificial ageing. Increased flexural strength was strongly linked with both polymerisation time and intensity [51].

Roughness decreased with more immersion in hot water. Roughness was unaffected by post-curing time when samples were subsequently submerged in boiling water. The mechanism for enhanced energy absorption as the temperature rises is responsible for the improvement in surface characteristics, particularly surface roughness, following placement in boiling water. The degree of monomer polymerisation has been linked to the surface characteristics of 3D-printed resins, and the increase in energy favours the polymerisation of unreacted monomers [52].

The polymerisation of 3D-printed PMMA resins is adversely affected by oxygen inhibition. Physical, mechanical, and biological properties are enhanced by limiting oxygen exposure after post-curing. In comparison to conventional polymerization, oxygen inhibition control has the following advantages: a higher degree of conversion (up to 12–42%); improved mechanical properties (higher flexural strength, flexural modulus, and microhardness, better fracture resistance, and surface smoothness); improved biocompatibility (less leaching of toxic compounds and unreacted monomers); improved colour stability (less staining and discolouration over time); and decreased solubility and water sorption. These can be accomplished by immersion in water, vacuum polymerisation, nitrogen atmosphere, and glycerin. There is no agreement on the optimal technique; glycerin and nitrogen work quite effectively. Standardised procedures and clinical validation should be the main focus of future studies [53].

Research has shown that increasing the postcuring temperature significantly improves the flexural properties, Vickers hardness, biocompatibility, and protein adsorption; increasing the postcuring time improves the flexural properties, Vickers hardness, and biocompatibility; and using a longer postcuring time at a low temperature delivers results comparable to using a shorter postcuring time at a higher postcuring temperature [54,55]. According to other research, surface roughness did not change with increasing post-curing time, whereas hardness increased [14]. While WR provides a simple output from a given experiment, SWR is known as an essential, standardised scientific metric. SWR transforms a condition-specific measurement into a fundamental material property, making it the critical parameter for ranking the long-term durability of dental restorative materials.

Regarding post-processing methods for 3D-printed materials, material III (with a longer postcuring time) shows relatively low Sa after wear, low wear rates, and Ra values that remain within the clinically smooth range, indicating stable, well-smoothed surfaces with limited material loss.

**H04:**  *Different post-processing protocols of the printed materials do not significantly influence the wear behaviour of the tested materials was rejected.*

The study limitations are related to: a two-body, constant-load in vitro setup, which cannot fully reproduce the complex, multi-directional, and intermittent loading and motion patterns of the oral environment; the use of a hard spherical antagonist and simplified lubricant; limited number and types of printed and milled interim materials; assessed short-term wear under fixed load and cycle number; the post-processing protocols did not cover the full clinical variability in post-curing time/temperature, oxygen inhibition control, and polishing techniques.

Given these limitations, additional research is needed to: implement more clinically relevant wear protocols, combining variable and multi-axial loading, complex motion, and ageing (thermocycling, brushing); systematically relate wear behavior to resin composition, microstructure, key mechanical properties; optimize and standardize 3D-printing post-processing protocols (washing, post-curing temperature/time, oxygen inhibition control, surface finishing) and quantify its impact on wear, roughness, hardness, and flexural properties.

## 5. Conclusions

The findings of this in vitro study provide new insights into the performance of contemporary dental interim restorative materials and have important implications for their clinical selection and application.

1. Wear statistics in this study demonstrated a consistent advantage for the printed resins and showed that the manufacturing method has a significant impact on the degree and rate of wear. Printed resins are a more suitable choice for long-term interim restoration.
2. Hardness has a central role in governing the wear performance of interim resin materials.
3. Optimised post-processing of printed materials, particularly a longer postcuring time, exhibits low wear and thus a high clinical potential. To provide optimised restorations, a longer postcuring time should be selected.

## Figures and Tables

**Table 1:** Materials used in the research.

Materials Group No.	Manufacturing Method	Material	Manufacturer	Composition/Structure	Post-Processing
I	3D Printed	Lumina	Dentona AG, Dortmund, Germany	Highly filled methacrylate resin, layer-wise structure, post-cured	Rinsed in pure isopropyl alcohol for 5 min Light curing 7 min/22 °C Mechanical polishing
II	3D Printed	Lumina	Dentona AG, Dortmund, Germany	Highly filled methacrylate resin, layer-wise structure, post-cured	Rinsed in pure isopropyl alcohol for 5 min Light curing 7 min/60 °C Mechanical polishing
III	3D Printed	Lumina	Dentona AG, Dortmund, Germany	Highly filled methacrylate resin, layer-wise structure, post-cured	Rinsed in pure isopropyl alcohol for 5 min Light curing 20 min/22 °C Mechanical polishing
IV	3D Printed	Lumina	Dentona AG, Dortmund, Germany	Highly filled methacrylate resin, layer-wise structure, post-cured	Rinsed in pure isopropyl alcohol 5 min Light curing 7 min/22 °C + glycerin Mechanical polishing
V	Milled	Telio CAD	Ivoclar Vivadent AG, Liechtenstein	High-conversion PMMA disc with compact, low-porosity matrix	Mechanical polishing
VI	Milled	Shaded PMMA	Dentsply Sirona, Bensheim, Germany	High-conversion PMMA disc with compact, low-porosity matrix	Mechanical polishing
VII	Milled	Copra Temp Symphony	Whitepeaks Dental Solutions GmbH, Hamminkeln, Germany	High-conversion PMMA disc with compact, low-porosity matrix	Mechanical polishing

**Table 2:** Mean values of volumetric loss (V), height of the vertical loss (h), specific wear rate (SWR) and wear rate (WR) with SD for worn specimens—all materials.

Material Group	V (mm^3^)	H (mm)	SWR ×10^−3^ mm^3^/(N·m)	WR (μm/1000 Cycles)
I	0.395 ± 0.12	0.022 ± 0.001	0.091 ± 108	3.7 ± 0.38
II	0.399 ± 0.09	0.022 ± 0.002	0.092 ± 0.433	3.694 ± 0.32
III	0.307 ± 0.07	0.020 ± 0.001	0.071 ± 0.255	3.252 ± 0.31
IV	0.459 ± 0.11	0.024 ± 0.002	0.106 ± 0.385	3.972 ± 0.23
V	2.975 ± 0.13	0.06 ± 0.003	0.69 ± 1.196	9.926 ± 0.34
VI	2.362 ± 0.1	0.054 ± 0.002	0.548 ± 1.174	8.995 ± 0.48
VII	1.573 ± 0.09	0.044 ± 0.003	0.365 ± 0.542	7.375 ± 0.22

**Table 3:** *p*-values the statistical Student *t*-Test between groups, in terms of SWR and WR.

	SWR	WR
I–II	0.930	0.977
I–III	0.026	0.009
I–IV	0.170	0.18
I–V	0.018	0.002
I–VI	0.003	<0.001
I–VII	<0.001	<0.001
II–III	0.076	0.089
II–IV	0.826	0.315
II–V	0.019	0.002
II–VI	0.003	<0.001
II–VII	<0.001	<0.001
III–IV	0.002	<0.001
III–V	0.016	0.001
III–VI	0.002	<0.001
III–VII	<0.001	<0.001
IV–V	<0.001	0.446
IV–VI	0.003	1
IV–VII	<0.001	0.027
V–VI	0.496	0.446
V–VII	0.123	0.052
VI–VII	0.034	0.027

**Table 4:** *p*-values of the statistical student *t* Test, between groups in terms of V and h.

	V	h
I–II	0.930	0.970
I–III	0.007	0.008
I–IV	0.176	0.180
I–V	0.006	0.001
I–VI	0.0007	0.0001
I–VII	0.01	0.01
II–III	0.094	0.089
II–IV	0.331	0.315
II–V	0.006	0.001
II–VI	0.0008	0.01
II–VII	0.01	0.01
III–IV	0.01	0.006
III–V	0.005	0.001
III–VI	0.000651	0.01
III–VII	0.01	0.01
IV–V	0.006765	0.002284
IV–VI	0.000963	0.01
IV–VII	0.01	0.01
V–VI	0.360	0.445
V–VII	0.058	0.445
VI–VII	0.034	0.026

**Table 5:** Mean values of microroughness with SD for polished and worn specimens (p = polished, w = worn).

Material Group	I	II	III	IV	V	VI	VII
Ra p	0.20 ± 0.02	0.14 ± 0.01	0.12 ± 0.02	0.09 ± 0.02	0.13 ± 0.02	0.13 ± 0.02	0.13 ± 0.02
Ra w	0.12 ± 0.01	0.13 ± 0.02	0.15 ± 0.01	0.07 ± 0.01	0.09 ± 0.01	0.11 ± 0.02	0.14 ± 0.01
Rz p	1.75 ± 0.26	1.09 ± 0.21	0.89 ± 0.01	0.90 ± 0.18	1.11 ± 0.21	1.04 ± 0.25	1.03 ± 0.18
Rz w	0.92 ± 0.19	1.15 ± 0.25	1.27 ± 0.18	0.86 ± 0.14	0.83 ± 0.23	0.93 ± 0.16	1.40 ± 0.19

**Table 6:** *p*-values—the statistical Student *t*-Test, in terms of Ra, Rz for polished and worn specimens.

p	I	II	III	IV	V	VI	VII
Ra p-w	<0.001	0.500	<0.001	<0.001	<0.001	0.002	0.084
Rz p-w	<0.001	0.458	0.001	0.616	0.006	0.078	0.002

**Table 7:** Mean values of nanoroughness parameters with SD for polished (p) and worn (w) specimens—all materials.

	I	II	III	IV	V	VI	VII
Sa p	18.2 ± 2.53	15.6 ± 2.67	11.18 ± 1.3	11.97 ± 1.52	5.99 ± 1.10	5.03 ± 0.41	3.90 ± 0.43
Sa w	7.35 ± 1.25	5.72 ± 1.29	5.15 ± 0.98	6.03 ± 1.46	3.71 ± 0.58	2.73 ± 0.66	2.26 ± 0.50
Sy p	148.30 ± 13.83	127.01 ± 12.02	95.62 ± 11.02	130.45 ± 27.60	52.03 ± 11.02	47.72 ± 12.93	43.06 ± 10.53
Sy w	57.53 ± 5.71	50.30 ± 4.56	42.63 ± 3.01	53.42 ± 5.96	29.67 ± 4.49	27.58 ± 2.92	17.09 ± 2.44

**Table 8:** *p*-values—the statistical Student *t*-Test in terms of Sa, Sq for polished and worn specimens.

p	I	II	III	IV	V	VI	VII
Sa p-w	0.012	0.004	0.018	0.624	0.004	0.003	0.511
Sq p-w	0.008	0.002	0.019	0.004	0.005	0.002	0.004

**Table 9:** Vickers hardness on samples for all materials.

Material Group	I	II	III	IV	V	VI	VII
HV	57.42 ± 5.74	49.53 ± 4.09	49.73 ± 3.89	92.37 ± 6.90	23.23 ± 3.11	23.95 ± 5.04	25.05 ± 3.04

**Table 10:** *p*-value—the statistical Student *t*-Test HV (Vickers Microhardness).

I–II	0.01
II–III	0.326
III–IV	0.01
IV–V	0.01
V–VI	0.06
VI–VII	0.005
I–III	0.01
II–IV	0.01
III–V	0.01
V–VII	0.01
I–IV	0.01
II–V	0.01
III–VI	0.01
I–V	0.01
II–VI	0.01

**Table 11:** Pearson correlations between SWR and Ra, Sa, HV.

Correlation Pair (Variables)	Pearson Coefficient r	Interpretation
SWR-Sa p	−0.45	Moderate negative correlation
SWR-Sa w	−0.35	Weak negative correlation
SWR-HV	−0.67	Strong negative correlation

## Data Availability

The raw data supporting the conclusions of this article will be made available by the authors on request.
